# On both sides of the arms race: The immune-eliciting and immune-suppressive powers of *Ralstonia solanacearum* effector PehC

**DOI:** 10.1093/plcell/koad107

**Published:** 2023-04-12

**Authors:** Bradley Laflamme

**Affiliations:** Assistant Features Editor, The Plant Cell, American Society of Plant Biologists, USA; Department of Molecular Genetics, University of Toronto, Toronto, ON M5S 1A1, Canada

From a macroscopic perspective, the arms race between plant pathogens and host immune systems can seem quite simple: pathogens try to evade perception by host receptors, and host receptors do their best to perceive pathogens. And yet, when we begin to decode how this perception—or lack thereof—is happening, it unveils a beautiful complexity. The field is replete with examples of receptors indirectly recognizing pathogen effector activity through decoy proteins and of effectors suppressing immunity by cleverly exploiting host proteins (Schreiber et al. 2021). Luckily for us, the breadth of pathogens and hosts out there means that we will likely never stop uncovering new and interesting molecular exchanges between hosts and pathogens.

In this issue, **Jingjing Ke, Wanting Zhu and colleagues** (Keet al. 2023) uncover multi-faceted activities of the PehC effector in *Ralstonia solanacearum*, a soil-borne bacterial pathogen of tomato (*Solanum lycopersicum*). Plant immune activation hinges on the perception of pathogens through receptors, which can recognize microbial signatures (e.g. flagellin), damage to plant tissues (e.g. cell wall degradation), or virulence activities of pathogens (e.g. effector proteins). To identify novel protein elicitors, Ke et al. challenged tomato roots with purified proteins from *R. solanacearum* culture. Remarkably, purified proteins were sufficient to activate several immune outputs (increased extracellular pH, reactive oxygen species bursts, MAP kinase cascade activation, and callose deposition) in the roots of both *R. solanacearum*-susceptible and -resistant tomato cultivars.

Using SDS-PAGE followed by LC-MS/MS, the authors identified PehC, a member of a class of pectin-degrading enzymes secreted by the pathogen's type II secretion system, as the most immunogenic protein in their cocktail. Immune elicitation by PehC could be achieved purely through its N-terminal region, rather than the middle region of the protein with known enzymatic activity. PehC also triggered similar immune outputs in the roots of other solanaceous plants and Arabidopsis (*Arabidopsis thaliana*) leaves, suggesting conserved or convergent strategies for perceiving PehC. However, immune elicitation was only half the story with PehC: even though purified PehC protein elicited immunity in tomato roots, a *ΔpehC* mutant was less virulent than the wild-type strain in colonizing the stems of tomato plants, suggesting that PehC's benefits to pathogenesis outweigh its immunogenic potential.

While studying the impact of PehC on the pectin-rich cell wall, they found that PehC was able to break down pectin-derived oligogalacturonic acids (OGs) into the monosaccharide galacturonic acid (GalA), thereby providing 2 major virulence benefits to *R. solanacearum*. Firstly, OGs are cell wall degradation products that can be perceived by host receptors to activate immunity (Ferrari 2013). In contrast, the GalA monomers, which PehC produces from OGs, did not activate any immune outputs, suggesting that the processing of OGs into GalA by PehC helps in evading host immunity. Secondly, the group found that GalA is an effective carbon source for *R. solanacearum* in a minimal medium. They further showed via LC-MS that GalA was rapidly produced in the xylem during *R. solanacearum* infection in a PehC-dependent manner. Given that the xylem is a nutrient-poor environment due to the mature vessel cells being dead (Zuluaga et al. 2013), PehC activity likely enhances virulence by producing a pathogen-accessible nutrient. These findings explain why an immune-eliciting effector would be conserved: it otherwise helps in immune evasion and nutrient acquisition (see Figure), 2 things for which every pathogen strives.

**Figure.**
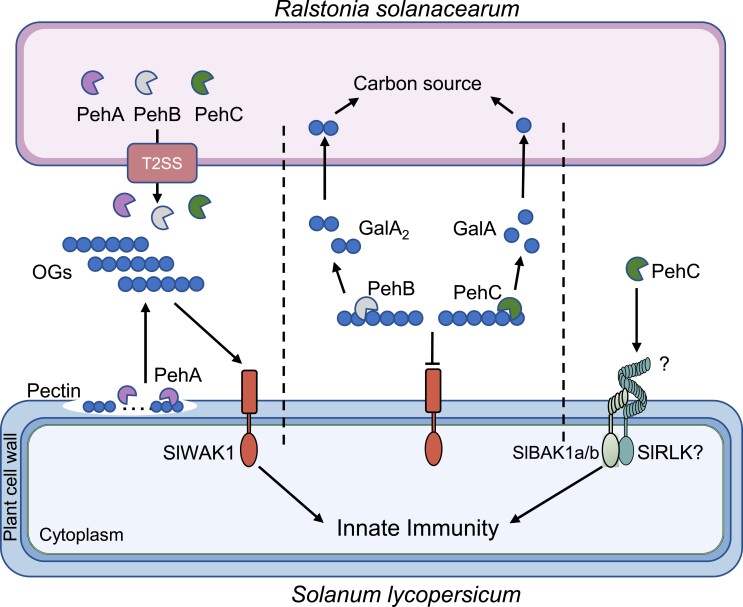
The roles of *R. solanacearum* PehA-C effectors during tomato infection. PehA produces OGs from pectin in the host cell wall, and PehB and PehC catabolize OGs into GalA dimers and monomers, respectively. Catabolizing OGs prevents immune receptors from perceiving OGs while also providing *R. solanacearum* with a carbon source. Additionally, PehC can be perceived by an unknown receptor. Reprinted from Ke et al. 2023, Figure 8.

With this study, Ke et al. identify an immune-eliciting effector by cleverly leaving the pathogen out of the equation. They then reintroduce the pathogen and show us why this elicitor still remains crucial for pathogenesis by studying its enzymatic activity. One might be curious, though, about how *R. solanacearum* deals with PehC immune elicitation during the early stages of infection. For example, PehC recognition might be suppressed by an unknown effector during the infection process, given that the suppression of effector recognition is a common output of effectors (Schreiber et al. 2021). So continues the back-and-forth between plants and microbes, between effectors and receptors: always different, always the same.
